# 2647. A Study of Risk Factors for Severe Influenza in South India

**DOI:** 10.1093/ofid/ofad500.2259

**Published:** 2023-11-27

**Authors:** Nidhi Prasad, Dantuluru Muralidhar, J Anitha, Chandana Acharya, Vandana KE, Vamsi A Surya, Chiranjay Mukhopadhyay, G Arun Kumar

**Affiliations:** Kasturba Medical College, Manipal, MAHE, Manipal, Karnataka, India; Department of infectious diseases, KMC Manipal , MAHE, PERAMPALLI UDUPI, Karnataka, India; Kasturba Medical College, Manipal, MAHE, Manipal, Karnataka, India; Kasturba Medical College, Manipal, MAHE, Manipal, Karnataka, India; Kasturba Medical College, Manipal, MAHE, Manipal, Karnataka, India; Kasturba Medical College, Manipal, MAHE, Manipal, Karnataka, India; Kasturba Medical College, Manipal, MAHE, Manipal, Karnataka, India; World Health Organization, Nepal, Manipal, Karnataka, India

## Abstract

**Background:**

Severe influenza infections are associated with high hospitalization and increased mortality. The risk factors for severe influenza A and B are not clearly studied in the Indian population. The aim of this study was to identify risk factors in severe influenza patients who had been admitted to a tertiary care referral hospital in South India.

**Methods:**

This was a prospective observational single center study conducted in admitted patients aged above 18 years who were confirmed to have influenza A and B by RTPCR from September 2019 to August 2021. Risk factors contributing to severe influenza were recorded. Exposure to risk factors was assessed and risk ratio/relative risk was calculated to determine strength of association. Statistical analysis was done by chi square test.

**Results:**

A total of 285 patients were diagnosed with influenza during the study period of which 129 (45%) had severe influenza. The distribution of types of influenza amongst severe influenza cases are shown in Figure 1.

Among all subtypes chronic kidney disease (CKD) (OR-4.98), Type 2 Diabetes (OR-3.14) had statistical significance with severe influenza. The risk factor analysis for severe influenza is shown in Table 1.

On analysis of the subtypes of influenza, it was found that, CKD and Type 2 Diabetes had significant association for severe H1N1. However chronic lung disease was identified as the only risk factor for severe H3N2 infection and Influenza B infection.

The Distribution of Types of Influenza Amongst Severe Influenza Cases

**Figure:**
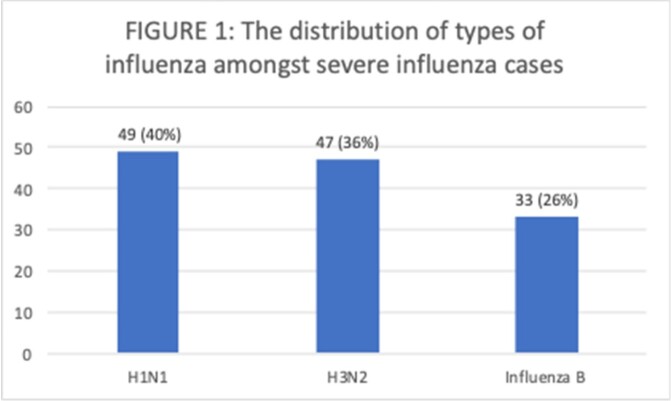

Risk Factor Analysis for Severe Influenza

**Figure:**
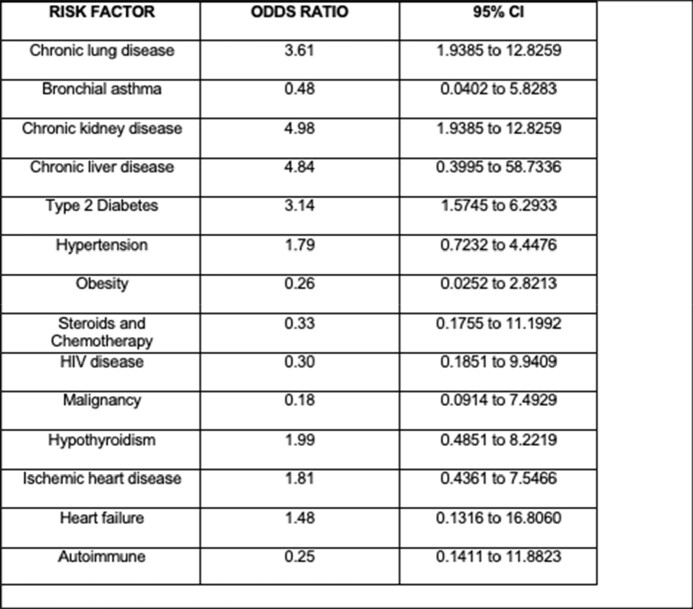

**Conclusion:**

CKD, Type 2 Diabetes and Chronic lung disease are major risk factors for severe influenza infections. Given the significance of these risk factors in the Indian population, early treatment and vaccination can significantly reduce mortality and morbidity.

**Disclosures:**

**All Authors**: No reported disclosures

